# EIF4A3-regulated circ_0087429 can reverse EMT and inhibit the progression of cervical cancer via miR-5003-3p-dependent upregulation of OGN expression

**DOI:** 10.1186/s13046-022-02368-4

**Published:** 2022-05-05

**Authors:** Meiqin Yang, Haoran Hu, Sufang Wu, Jianyi Ding, Bo Yin, Baoyou Huang, Fang Li, Xiaoqing Guo, Lingfei Han

**Affiliations:** 1 Department of Gynecology, Shanghai First Maternity and Infant Hospital, School of Medicine, Tongji University, Shanghai, 200092 China; 2 Department of Obstetrics and Gynecology, Shanghai General Hospital, Shanghai Jiao Tong University School of Medicine, Shanghai, 201620 China; 3 Department of Gynecology, the First Affiliated Hospital of Wenzhou Medical University, Wenzhou Zhejiang, 325027 China; 4 Department of Gynecology, Shanghai East Hospital, School of Medicine, Tongji University, Shanghai, 200120 China

**Keywords:** Circ_0087429, Cervical cancer, MiR-5003-3p, OGN, Epithelial to mesenchymal transition, EIF4A3

## Abstract

**Background:**

Circular RNAs (circRNAs) are noncoding RNAs with stable structures with high expression and tissue-specific expression. Studies have shown that circRNA dysregulation is closely related to the progression of tumours. However, the function and regulatory mechanism of most circRNAs in cervical cancer are still unclear.

**Methods:**

CircRNAs related to cervical cancer were screened through the Gene Expression Omnibus (GEO) database. qRT-PCR was used to verify the expression of circ_0087429 in cervical cancer tissues and cells. Then, in vivo and in vitro experiments were conducted to evaluate the role of circ_0087429 in the progression of cervical cancer. The role of the circ_0087429/miR-5003-3p/osteoglycin (OGN) axis in the epithelial to mesenchymal transition (EMT) was confirmed by rescue experiments, fluorescence in situ hybridization, luciferase reporter assays, immunofluorescence staining and western blotting. The inhibitory effect of Eukaryotic initiation factor 4A-III (EIF4A3) on the biogenesis of circ_0087429 was verified by RNA immunoprecipitation (RIP) assays and qRT-PCR.

**Results:**

circ_0087429 is significantly downregulated in cervical cancer tissues and cells and negatively correlated with International Federation of Gynecology and Obstetrics (FIGO) staging and lymphatic metastasis in cervical cancer patients. circ_0087429 can significantly inhibit the proliferation, migration, invasion and angiogenesis of cervical cancer in vitro and tumour growth and metastasis in vivo. OGN is significantly downregulated in cervical cancer tissues and cells. circ_0087429 can upregulate the expression of OGN by competitively binding with miR-5003-3p, thereby reversing EMT and inhibiting the progression of cervical cancer. EIF4A3 can inhibit circ_0087429 expression by binding to its flanking regions.

**Conclusions:**

As a tumour suppressor, circ_0087429 regulated by EIF4A3 can reverse EMT and inhibit the progression of cervical cancer through the miR-5003-3p/OGN axis. It is expected to become a potential target for the treatment of cervical cancer.

**Supplementary Information:**

The online version contains supplementary material available at 10.1186/s13046-022-02368-4.

## Introduction

Cervical cancer is the fourth most common cancer in the world and poses a serious threat to women's health [1]. Due to the increase in human papilloma virus (HPV) infections and the confusion regarding screening procedures, the incidence and mortality of cervical cancer have increased, while the age of onset has decreased [2]. A preventive vaccine for cervical cancer is currently on the market, but it is not effective for women with cervical precancerous lesions or cervical cancer [3]. Therefore, it is particularly important to further explore the molecular mechanism of the occurrence and development of cervical cancer and to actively screen for possible new specific molecular targets for the treatment of cervical cancer.

Circular RNA (circRNA) is a type of noncoding RNA with a closed-loop structure. Its structure is stable and not easily degraded by exonuclease due to the absence of 5' caps and 3' tails [4]. With the advancement of gene sequencing and bioinformatics technology in recent years, a large number of circRNAs have been found to be involved in various biological processes, such as cancer proliferation, migration, invasion and drug resistance [5, 6]. The mechanism of action of circRNA is also complex and diverse. CircRNAs can be used as scaffolds for the assembly of protein complexes [7]; circRNAs can regulate gene transcription and RNA splicing [8]; circRNAs containing internal ribosome entry site elements and open reading frames can also be translated into proteins or polypeptides [9]. However, the most common mechanism of action of circRNA in cancer is the competitive endogenous RNA (ceRNA) mechanism. Studies have found that most circRNA sequences contain miRNA binding sites, which allow them to interact with miRNAs. This interaction prevents the binding of miRNA and mRNA, and thus, a circRNA-miRNA-mRNA functional network is formed [10]. For example, in pancreatic ductal adenocarcinoma, circEYA3 induces energy production through the miR-1294/c-Myc axis to promote tumour progression [11]; circCCDC9 can regulate the expression of CAV1 by adsorbing miR-6792-3p to inhibit the occurrence and development of gastric cancer [12]. In cervical cancer, the role of some circRNAs, such as circTPCN [13] and circZFR [14], has been reported. However, the functions of most circRNAs have not yet been elucidated.

MicroRNAs (miRNAs) are endogenous short-stranded noncoding RNAs of nearly 20 nucleotides. They can inhibit translation and promote mRNA degradation [15]. The abnormal regulation of miRNA is closely related to tumorigenesis. Changes in miRNA expression can induce a series of cascading reactions and feedback pathways [16]. The role and related pathways of miR-5003-3p have not been well reported. Only Kwak et al.'s studies have shown that miR-5003-3p in breast cancer can promote the stability of snail by targeting E-cadherin and MDM2 and then promote the metastasis of breast cancer through the epithelial to mesenchymal transition (EMT) pathway [17]. There is no report on the role of miR-5003-3p in cervical cancer.

Small leucine-rich proteoglycans (SLRPs) are secreted by a variety of cells, are mainly located in the extracellular matrix, and can participate in various biological processes, such as signal transduction, cell adhesion, and DNA repair [18, 19]. Osteoglycin (OGN) is a type of SLRP that has been found to be downregulated in many different cancers, including cervical cancer, breast cancer, and colon cancer [20–22]. The specific role and regulatory mechanism of OGN in cervical cancer are still unknown.

In this study, we first discovered that circ_0087429 was significantly downregulated in cervical cancer. Subsequently, its clinical significance in cervical cancer was discussed, and its effect and regulatory mechanism were studied through in vivo and in vitro experiments. circ_0087429 can be used as a molecular sponge of miR-5003-3p to reverse EMT by regulating the expression of the target gene OGN, thereby inhibiting the occurrence and development of cervical cancer. EIF4A3 can inhibit the biogenesis of circ_0087429. This research provides a potential target for the pathogenesis and treatment of cervical cancer.

## Materials and methods

### Patient tissue samples and cell lines

This study included 44 patients with cervical cancer who were treated at the Shanghai First Maternity and Infant Hospital from January to December 2017. None of the patients received chemotherapy or radiotherapy before biopsy or surgery. After surgical resection, samples of cervical cancer tissues and matched adjacent tissues were immediately frozen in liquid nitrogen and then transferred to a -80 °C refrigerator for stable storage until use. This study was approved by the Ethics Committee of the Shanghai First Maternity and Infant Hospital. All the included patients signed informed consent forms. The histopathological and clinical data came from pathology reports and medical records.

A human cervical immortalized squamous cell line (Ect1/E6E7) and human cervical cancer cell lines (HeLa, SiHa and CaSki) were provided by the American Type Culture Collection (ATCC, USA). DMEM (Gibco, USA) was used to cultivate HeLa and SiHa cells, and RPMI-1640 medium (Gibco, USA) was used to cultivate CaSki cells. Ten percent foetal bovine serum (Gibco, USA) was added to the above two types of media. Ect1/E6E7 cells were cultured in keratinocyte serum-free medium with 0.1 ng/ml human recombinant EGF, 0.05 mg/ml bovine pituitary extract and 44.1 mg/L calcium chloride. All cell lines were cultured at 37 °C in a humidified incubator containing 5% CO^2^.

### Quantitative real-time polymerase reaction (qRT-PCR)

TRIzol reagent was used to extract total RNA from the tissue or cell line according to the manufacturer's instructions. For circRNA and mRNA, the PrimeScript RT reagent Kit (Takara, Japan) was used to synthesize total RNA into cDNA, and TB Green Premix Ex Taq II (Takara, Japan) was used for cDNA amplification. For miRNA, the microRNA Reverse Transcription Kit (EZBioscience, USA) was used for reverse transcription, and Probe qPCR Master Mix for microRNA (EZBioscience, USA) was used for cDNA expansion. β-Actin, GAPDH and U6 were used as internal controls. The expression of each gene was normalized to that of the internal control and quantified using the 2^−ΔΔCT^ method. Related primer sequences are shown in Additional file 1: Table S1.

### Cell transfection and stable transfection strain construction

Circ_0087429 was overexpressed by pcDNA5.1-ciR integrated with the full-length sequence of circ_0087429 (Geneseed, China). OGN and EIF4A3 was overexpressed by PGMLV-6395 integrated with the full-length sequence of OGN or EIF4A3 (Genomeditech, China). According to the manufacturer’s protocol, Lipofectamine 3000 Reagent (Thermo Fisher Scientific, USA) was used for transfection. After successful transfection, cell lines were screened with 2 μg/ml puromycin for 30 days. In addition, the silencing of circ_0087429, OGN and EIF4A3 were achieved by si-circ_0087429, si-OGN and si-EIF4A3 respectively, and si-NC was a negative control. The overexpression and inhibition of miR-5003-3p were achieved by miR-5003-3p mimics and miR-5003-3p inhibitors, and NC mimics and NC inhibitors were used as controls (provided by Genomeditech, China). The above oligonucleotide sequences are shown in Additional file 1: Table S2.

### RNase R treatment

Two micrograms of total RNA with or without 5 U/μg RNase R (Epicentre Technologies, USA) was incubated at 37 °C for 30 min. Then, qRT-PCR was used to detect the expression of circ_0087429 and the parental gene SPIN1.

### Actinomycin D assays

Cells were seeded in six-well plates (4 × 105 cells per well). Twenty-four hours later, 2 μg/ml actinomycin D (Sigma, USA) was added to the cells. Then, total RNA was collected at the specified time, and the expression of circ_0087429 and SPIN1 was detected by qRT-PCR. The half-life was the time required for the RNA level to reach 50% of that at 0 h.

### Nucleocytoplasmic fractionation

Separation of cytoplasmic and nuclear RNA in SiHa and HeLa cells was carried out using the Cytoplasmic and Nuclear RNA Purification Kit (Norgenbiotek Corporation, Canada). qRT-PCR was used to detect the relative expression levels of circ_0087429 and SPIN1. U6 was used as the internal reference for the nuclear fraction, and GAPDH was used as that for the cytoplasmic fraction.

### Fluorescence in situ hybridization (FISH)

FAM-labelled circ_0087429 and Cy3-labelled miR-5003-3p were used to observe the cellular localization of the two. According to the manufacturer's instructions, circ_0087429 and miR-5003-3p probes were used for hybridization overnight, and then the nuclei were counterstained with DAPI. The pictures were acquired by a Zeiss LSM710 laser scanning confocal microscope (Zeiss Instrument Inc., Germany).

### Cell Counting Kit-8 (CCK-8) assay

CCK-8 reagent (Dojindo, Japan) was used to evaluate the proliferation ability of cervical cancer cells. Cells were seeded in 96-well plates at 1 × 10^3^ cells per well. After culturing for 0, 24, 48, 72, and 96 h, CCK-8 solution (10 µL) was added to each well, incubated for 2 h in an incubator, and then a spectrophotometer was used to evaluate the absorbance at 450 nm.

### 5-Ethynyl-20-deoxyuridine (EdU) Assay

The Cell-Light EdU DNA cell proliferation kit (RiboBio, China) was used for EdU detection. Cells were incubated with EdU for 2 h and then fixed with 4% paraformaldehyde, followed by dyeing and sealing with Apollo dye solution and Hoechst 33,342. An inverted fluorescence microscope (Carl Zeiss, Germany) was used to take pictures to evaluate the proportion of EdU-positive cells.

### Colony formation assay

The cells to be tested were seeded in a 6-well plate at 800 cells per well. After 2 weeks of culture, the cells were fixed with 4% paraformaldehyde for 15 min and stained with 0.5% crystal violet solution for 15 min. Finally, the colonies were counted.

### Wound healing assay

The cells were cultured in a 6-well plate to 90% confluence. A 200-µl pipette tip was used to create a scratch of the same width. A microscope was used to acquire images immediately. The cells were then incubated in serum-free medium for 24 h and 48 h and photographed again. The wound width was recorded at each time point.

### Transwell invasion assay

The transwell chamber (Corning, USA) was paved with matrigel mix (BD Biosciences, USA) for invasion assays. According to the manufacturer's protocol, 200 µl of the cell suspension was placed in serum-free medium in the upper chamber, and 20% FBS medium was added to the lower chamber. After 24 h of incubation, cotton swabs were used to remove the cells inside the upper chamber. The cells that invaded the bottom of the membrane were fixed and stained. A microscope was used to take pictures and then evaluate the number of cells with ImageJ software.

### Tube formation assay

HUVECs were plated in 96-well plates coated with Matrigel (Corning, USA) at a density of 4 × 104 cells per well. After incubating for 3–4 h in the incubator, calcein AM (Sigma, USA) was added to the plate. A fluorescence microscope (Carl Zeiss, Germany) was used to take pictures and then analyse the tube-forming ability of each group of cells.

### Western blotting

One percent PMSF added to RIPA lysis buffer (Beyotime, China) was used to extract total proteins. The same amounts of protein were separated by 10% SDS-PAGE, transferred to PVDF membranes (Millipore, USA), and then sealed with skim milk. The blots were incubated with the primary antibody at 4 °C for 12 h and then with the secondary antibody at room temperature for 1 h. An electrochemiluminescence kit (Millipore, USA) was used for the visualization of protein signals. β-Actin was the internal control. Primary antibodies were anti-E-cadherin, anti-N-cadherin, anti-Claudin-1, anti-Vimentin, anti-Snail, anti-OGN, anti-MMP2 and anti-EIF4A3 from Abcam (Cambridge, UK).

### Luciferase reporter assay

The sequences that bind to miR-5003-3p in the circ_0087429 region and the OGN-3’UTR and their corresponding mutant sequences were synthesized and inserted into the luciferase reporter vector GM-1013FL02 and named circ_0087429-WT, circ_0087429-MUT, OGN-WT and OGN-MUT, respectively (Genomeditech, China). These plasmids were cotransfected with miR-5003-3p mimics or mimics NC. Then, according to the manufacturer's protocol, the relative luciferase activity was checked with the Pierce Renilla-Firefly Luciferase Dual Assay Kit (Thermo Fisher Scientific, USA).

### Cell immunofluorescence (IF) staining

A 4% formaldehyde solution was used to fix the cells for 15 min. Triton X-100 (0.2%) was used to permeabilize the cell membrane for 30 min. Then, the cells were blocked in blocking solution at room temperature for 1 h, and the primary antibodies were added and incubated overnight at 4 °C. Then, the fluorophore-tagged secondary antibodies were incubated for 1 h at room temperature. After that, the nuclei were counterstained with DAPI for 15 min and photographed with a confocal microscope.

### RNA immunoprecipitation (RIP) assay

RIP assay was performed using an EZMagna RIP kit (Millipore, USA) according to the manufacturer's protocol. The magnetic beads were incubated with anti-EIF4A3 antibody or IgG negative control antibody. Cell lysates were then incubated with the corresponding antibody-coated beads. Finally, the immunoprecipitated RNA was extracted and detected by qRT-PCR.

### Immunohistochemistry (IHC) examination

Tissue sections were deparaffinized with xylene and dehydrated with a graded alcohol series. Afterwards, the slices were treated with 3% hydrogen peroxide and pressure-cooked for antigen retrieval. Then, the slices were incubated with anti-OGN, anti-E-cadherin, anti-N-cadherin, anti-CD31 and anti-Ki67 primary antibodies from Abcam (Cambridge, UK) at 4 °C overnight and then incubated with the appropriate secondary antibodies for 1 h at room temperature. Finally, the sections were treated with 3'-diaminobenzidine tetrahydrochloride and counterstained with haematoxylin.

### Xenograft assay

All animal care and experiments were conducted in accordance with the guidelines of the National Institutes of Health and approved by the Animal Care Committee of Tongji University. We chose 4-week-old female BALB/c nude mice for tumour xenografts experiments. In tumour growth assay in vivo, HeLa cells stably transfected with pcDNA5.1-NC or pcDNA5.1-circ_0087429 were injected subcutaneously into the upper back of nude mice (1 × 10^7^, 100 μl). The size of the tumours was measured with a calliper every week. The tumour volume was calculated as length x width^2^ × 0.5. Twenty-eight days after the injection, the mice were euthanized, and the tumours were dissected for further analysis. In tumour metastasis assay in vivo, HeLa cells stably transfected with pcDNA5.1-NC or pcDNA5.1-circ_0087429 were injected into nude mice through the tail vein (5 × 10^6^, 150 μl). After five weeks, the mice were euthanized, and the livers and lungs were dissected, embedded in paraffin, and finally validated by haematoxylin and eosin (H&E) staining.

### Statistical analysis

Statistical analyses were performed using SPSS 22.0 (IBM, SPSS, Chicago, USA) and GraphPad Prism 9.1.0 (GraphPad, La Jolla, CA, USA). Student's t test (two-tailed) was used to compare the differences between the two groups, and one-way ANOVA was used to compare the differences among multiple groups. Pearson correlation analysis was used to analyse the correlations. The results of quantitative data are expressed as the mean ± SD. All experiments were repeated at least 3 times. For all the above analysis results, *p* < 0.05 was considered to indicate a significant difference.

## Results

### The expression and clinical significance of circ_0087429 in cervical cancer

**Fig. 1:**
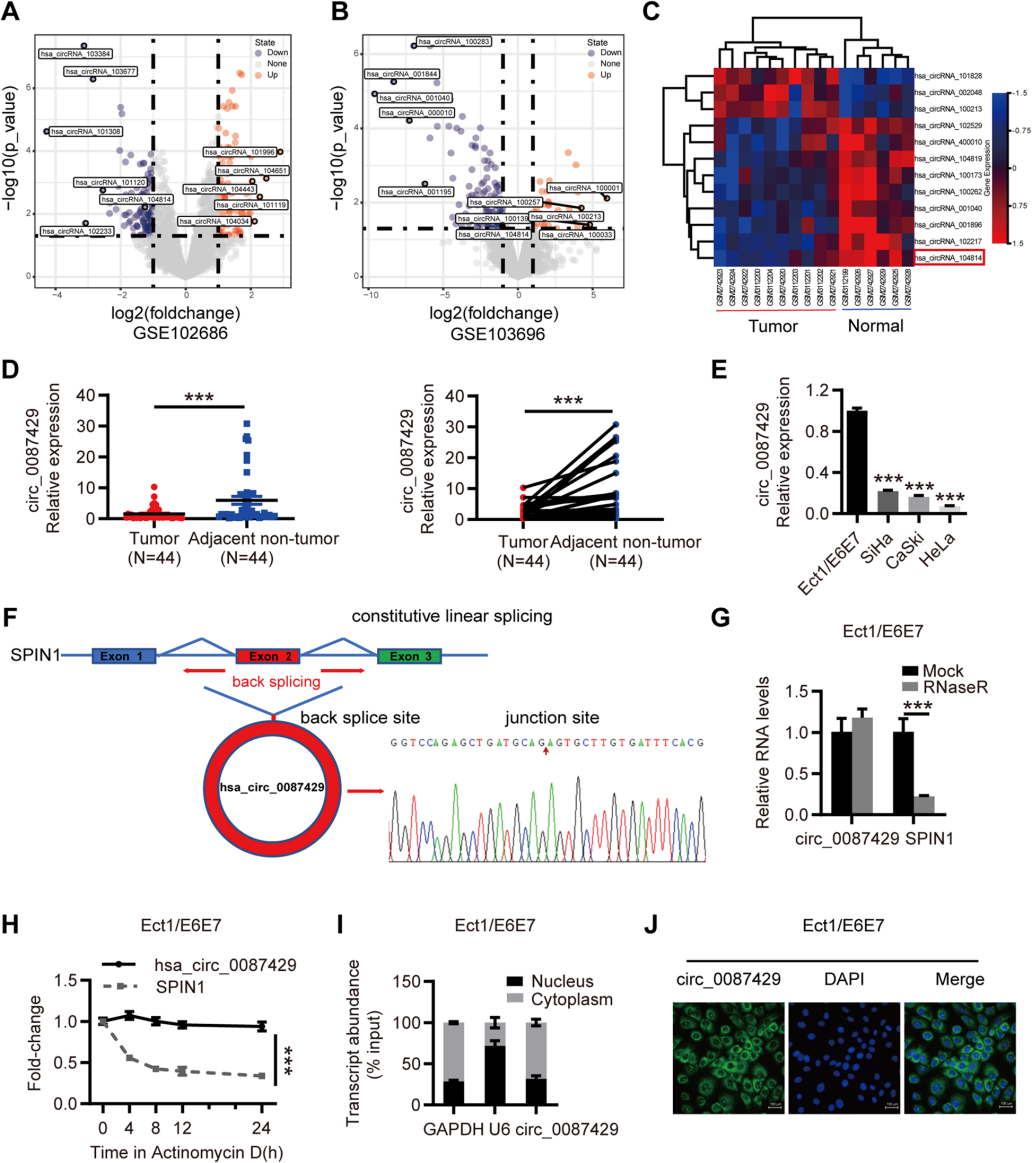
The expression and characteristics of circ_0087429 in cervical cancer. **a.** Differentially expressed circRNAs between cervical cancer and adjacent tissue samples in the GSE102686 dataset. **b.** Differentially expressed circRNAs between cervical cancer and normal cervical epithelial cell lines in the GSE103696 dataset. **c.** Heatmap of circRNAs with significant differential expression in two datasets (GSE102686 and GSE103696). circ_0087429 (hsa_circRNA_104814) is marked with a red box. **d.** Differential expression of circ_0087429 in 44 pairs of cervical cancer and adjacent tissue samples. **e.** Differential expression of circ_0087429 between cervical cancer and normal cervical epithelial cell lines. **f.** The Sanger sequencing results showed that circ_0087429 was formed by head-to-tail splicing of the second exon of the parental gene SPIN1. **g.** The expression of circ_0087429 and SPIN1 after RNaseR treatment was detected by qRT-PCR. **h.** The expression changes in circ_0087429 and SPIN1 at different time points after adding actinomycin D were determined by qRT-PCR. **i.** A subcellular fractionation assay was used to detect the relative expression of circ_0087429 in the cytoplasm and nucleus. U6 was used as the internal reference for the nuclear fraction, and GAPDH was used as that for the cytoplasmic fraction. **j.** FISH staining confirmed the expression of circ_0087429 in the cytoplasm. Scale bar, 100 μm. ***p < 0.001

First, we screened the differentially expressed circRNAs between cervical cancer and normal cervical samples in circRNA microarray datasets in the Gene Expression Omnibus (GEO) database. The GSE102686 dataset contained 5 pairs of cervical cancer and adjacent tissue samples, and the GSE113696 dataset contained 1 normal cervical epithelial cell line, HcerEpic, and 5 cervical cancer cell lines, HeLa, CaSki, SiHa, C-33A, SW756. Both groups of microarray datasets showed that hsa_circRNA_104814 (hsa_circ_0087429) was downregulated by more than onefold in cervical cancer versus adjacent tissues (Fig. 1a-c). To further verify this expression difference, we collected 44 pairs of cervical cancer and adjacent tissue samples. The qRT-PCR results showed that circ_0087429 was significantly downregulated in cervical cancer tissues, which was consistent with the microarray results (Fig. 1d). The relationship between circ_0087429 and the clinicopathological parameters of cervical cancer patients is shown in Table 1. Compared with that in stage I cervical cancer patients, the expression of circ_0087429 in stage II patients was significantly reduced; the expression of circ_0087429 was reduced in patients with lymphatic metastasis compared with non-lymphatic metastasis.

**Table 1:** Correlations between the expression of circ_0087429 and OGN and clinicopathological parameters of cervical cancer patients

**Characteristics**	**Cases**	**circ_0087429 expression**			**OGN expression**		
		**Low(22)**	**High(22)**	***p***	**Low(22)**	**High(22)**	***p***
Age (years)				0.531			0.531
< 45	16	9	7		9	7	
≥ 45	28	13	15		13	15	
FIGO stage				0.042^*^			0.176
Ib	32	13	19		14	18	
IIa-IIb	12	9	3		8	4	
Tumour size(cm)				0.099			0.003^*^
< 4	31	13	18		11	20	
≥ 4	13	9	4		11	2	
Lymph node invasion				0.031^*^			0.150
Absent	34	14	20		15	19	
Present	10	8	2		7	3	
Stromal invasion				0.099			0.741
< 1/2	13	4	9		6	7	
≥ 1/2	31	18	13		16	15	

FIGO International Federation of Gynecology and Obstetrics; ^*^ *P* < 0.05

Next, we detected the expression of circ_0087429 in the normal cervical epithelial cell line Ect1/E6E7 and cervical cancer cell lines HeLa, SiHa and CaSki. The expression of circ_0087429 was significantly reduced in cervical cancer cell lines, and the expression of circ_0087429 was the lowest in HeLa cells (Fig. 1e).

### Features and location of circ_0087429

The Sanger sequencing results showed that circ_0087429 originates from the SPIN1 gene located on chromosome 9 and is formed by the splicing of the head and tail of the second exon (Fig. 1f). To further verify the ring structure of circ_0087429, we used RNase R and actinomycin D to verify the stability of circ_0087429. The results showed that compared with SPIN1, circ_0087429 was resistant to RNase R and actinomycin D treatment (Fig. 1g-h). In addition, we detected the cellular location of circ_0087429 by subcellular grade analysis and FISH. We confirmed that circ_0087429 was mainly distributed in the cytoplasm (Fig. 1i-j). The above results indicated that circ_0087429 may be involved in posttranscriptional regulation.

### Circ_0087429 inhibits the proliferation, migration, invasion and angiogenesis of cervical cancer in vitro

**Fig. 2:**
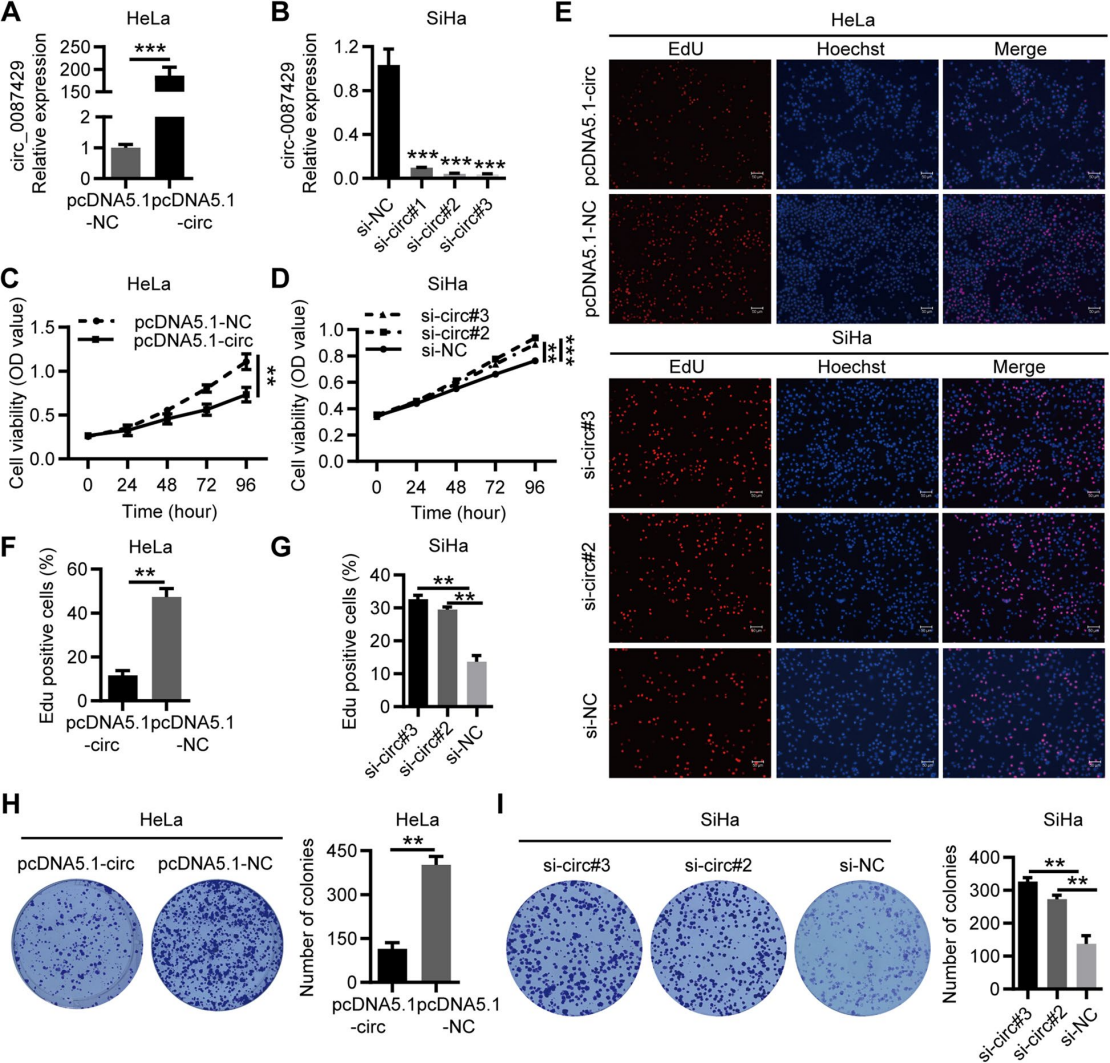
circ_0087429 inhibits the proliferation of cervical cancer in vitro. **a.** The expression of circ_0087429 in HeLa cells stably transfected with pcDNA5.1-circ_0087429 was detected by qRT-PCR. **b.** The expression of circ_0087429 in SiHa cells treated with siRNA was detected by qRT-PCR. **c-g.** CCK-8 assays and EdU assays were used to detect changes in cell proliferation after overexpression or knockdown of circ_0087429. Scale bar, 50 μm. **h-i.** Colony formation assays were used to detect the proliferation ability of cells after overexpression or knockdown of circ_0087429. **p < 0.01, ***p < 0.001

**Fig. 3:**
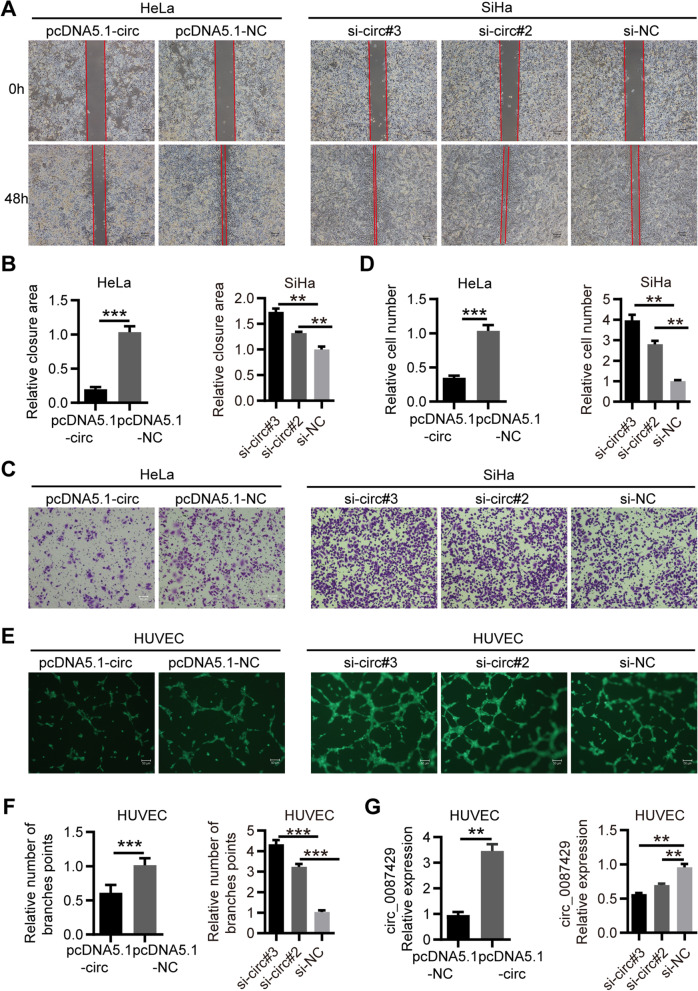
circ_0087429 inhibits migration, invasion and angiogenesis of cervical cancer in vitro. **a-b.** Wound healing assays determined the migration ability of cells after overexpression or knockdown of circ_0087429. Scale bar, 100 μm. **c-d.** Transwell invasion assays detected the invasion ability of cells after overexpression or knockdown of circ_0087429. Scale bar, 50 μm. **e–f.** After HUVECs were cocultured with circ_0087429-overexpressing or circ_0087429-knockdown cells, the tube-forming ability was detected. Scale bar, 50 μm. **g.** The expression of circ_0087429 in HUVECs after coculture with circ_0087429 overexpression or knockdown cells was detected by qRT-PCR. **p < 0.01, ***p < 0.001

To explore the biological function of circ_0087429, we constructed an overexpression lentiviral vector for circ_0087429 and established a stable HeLa cell line overexpressing circ_0087429 (Fig. 2a). In addition, three siRNAs targeting circ_0087429 were constructed to knock down circ_0087429 in SiHa cells (Fig. 2b). The results of CCK-8 assays, EdU assays and colony formation assays showed that overexpression of circ_0087429 inhibited the proliferation of cervical cancer cells, while circ_0087429 knockdown promoted their proliferation (Fig. 2c-i). Wound healing and Transwell invasion assays showed that the overexpression of circ_0087429 inhibited the migration and invasion of cervical cancer cells, while circ_0087429 knockdown showed the opposite effects (Fig. 3a-d). HUVEC tube formation experiments showed that when HUVECs were cocultured with cervical cancer cells with circ_0087429 overexpression or knockdown, the tube-forming ability of HUVECs was reduced or enhanced (Fig. 3e-f). To this end, we assessed the expression change of circ_0087429 in HUVECs after coculture, and the results showed that the expression of circ_0087429 in HUVECs increased after coculture with cervical cancer cells overexpressing circ_0087429, and the expression of circ_0087429 decreased after coculture with cervical cancer cells with circ_0087429 knockdown (Fig. 3g). In short, these data suggested that circ_0087429 can inhibit the proliferation, migration and invasion of cervical cancer cells and alter their angiogenesis ability by affecting the expression of circ_0087429 in peripheral vascular endothelial cells.

### Circ_0087429 inhibits the progression of cervical cancer by targeting miR-5003-3p

**Fig. 4:**
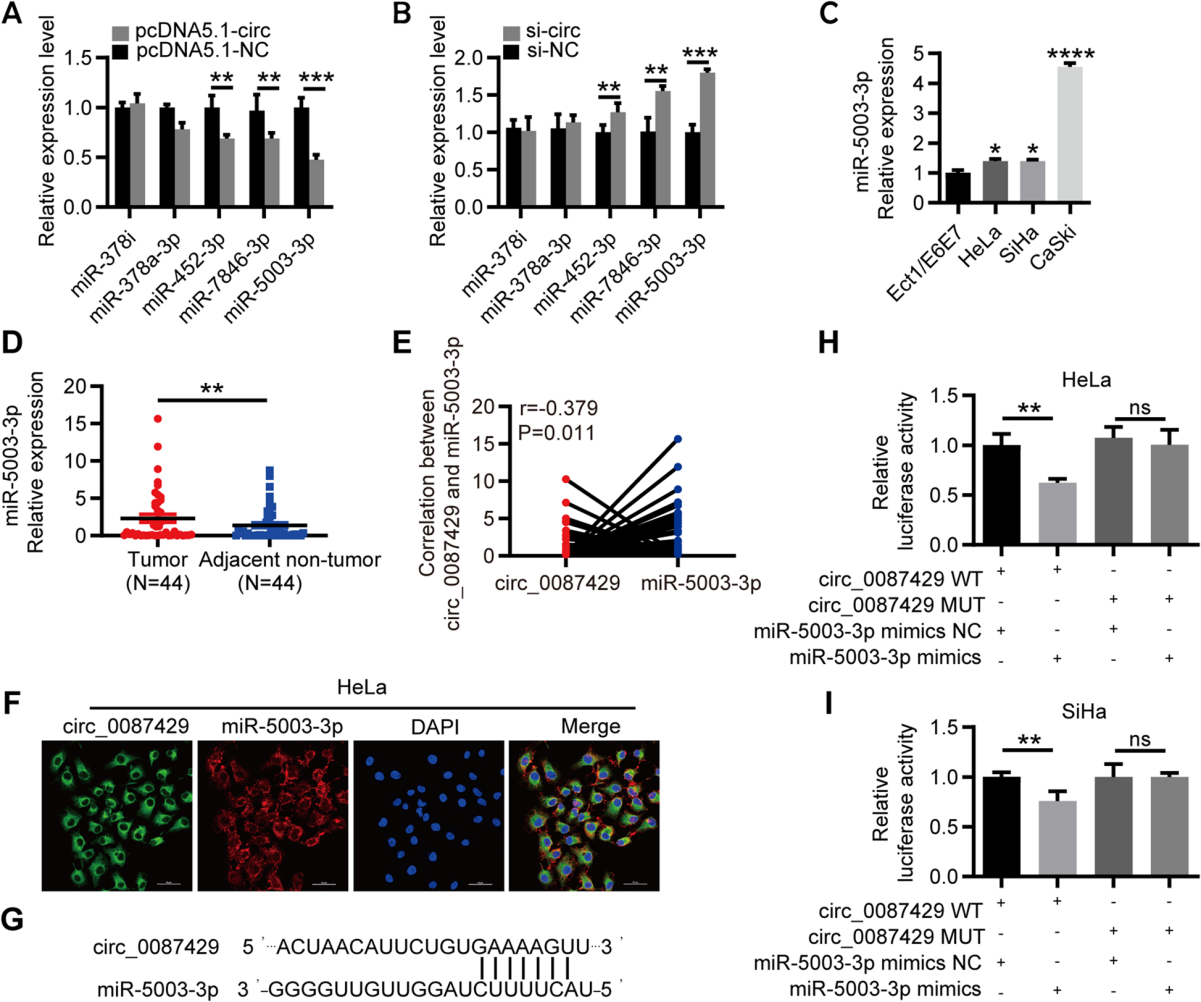
circ_0087429 can competitively bind with miR-5003-3p. **a-b.** The expression changes in candidate miRNAs after circ_0087429 overexpression or knockdown were determined by qRT-PCR. **c.** The differential expression of miR-5003-3p between cervical cancer and normal cervical epithelial cell lines was detected by qRT–PCR. **d.** The differential expression of miR-5003-3p between cervical cancer and adjacent tissue samples was detected by qRT–PCR. **e.** Spearman’s correlation analysis confirmed the correlation between circ_0087429 and miR-5003-3p expression in cervical cancer tissue samples. **f.** The colocalization of circ_0087429 and miR-5003-3p in cells was detected by FISH staining. Scale bar, 50 μm. **g.** The binding sites of circ_0087429 and miR-5003-3p were predicted by circBank. **h-i.** A luciferase reporter assay was used to examine the relative luciferase activity of the circ_0087429WT and circ_0087429MUT. **p < 0.01, ***p < 0.001, NS means no statistical significance

Current research has shown that circRNAs mainly regulate mRNA expression through competitive binding with miRNAs. Given that circ_0087429 is mainly located in the cytoplasm and exhibits obvious stability, we used the miRNA target prediction tool circBank [23] to predict the potential targets of circ_0087429 and selected 5 candidate miRNAs for further verification. The qRT-PCR results showed that miR-5003-3p was significantly downregulated after overexpression of circ_0087429, and when the expression of circ_0087429 was reduced, the expression of miR-5003-3p was significantly increased (Fig. 4a-b). miR-5003-3p was significantly upregulated in cervical cancer and negatively correlated with the expression of circ_0087429 (Fig. 4c-e). The FISH results showed that miR-5003-3p and circ_0087429 colocalized in the cytoplasm (Fig. 4f). Then, based on the binding sites of circ_0087429 and miR-5003-3p, we constructed circ_0087429-WT and circ_0087429-MUT plasmids. The results of the dual-luciferase reporter assay showed that overexpression of miR-5003-3p significantly reduced the luciferase activity of circ_0087429-WT but did not affect the luciferase activity of circ_0087429-MUT (Fig. 4g-i). These results confirmed that circ_0087429 can interact with miR-5003-3p.

**Fig. 5:**
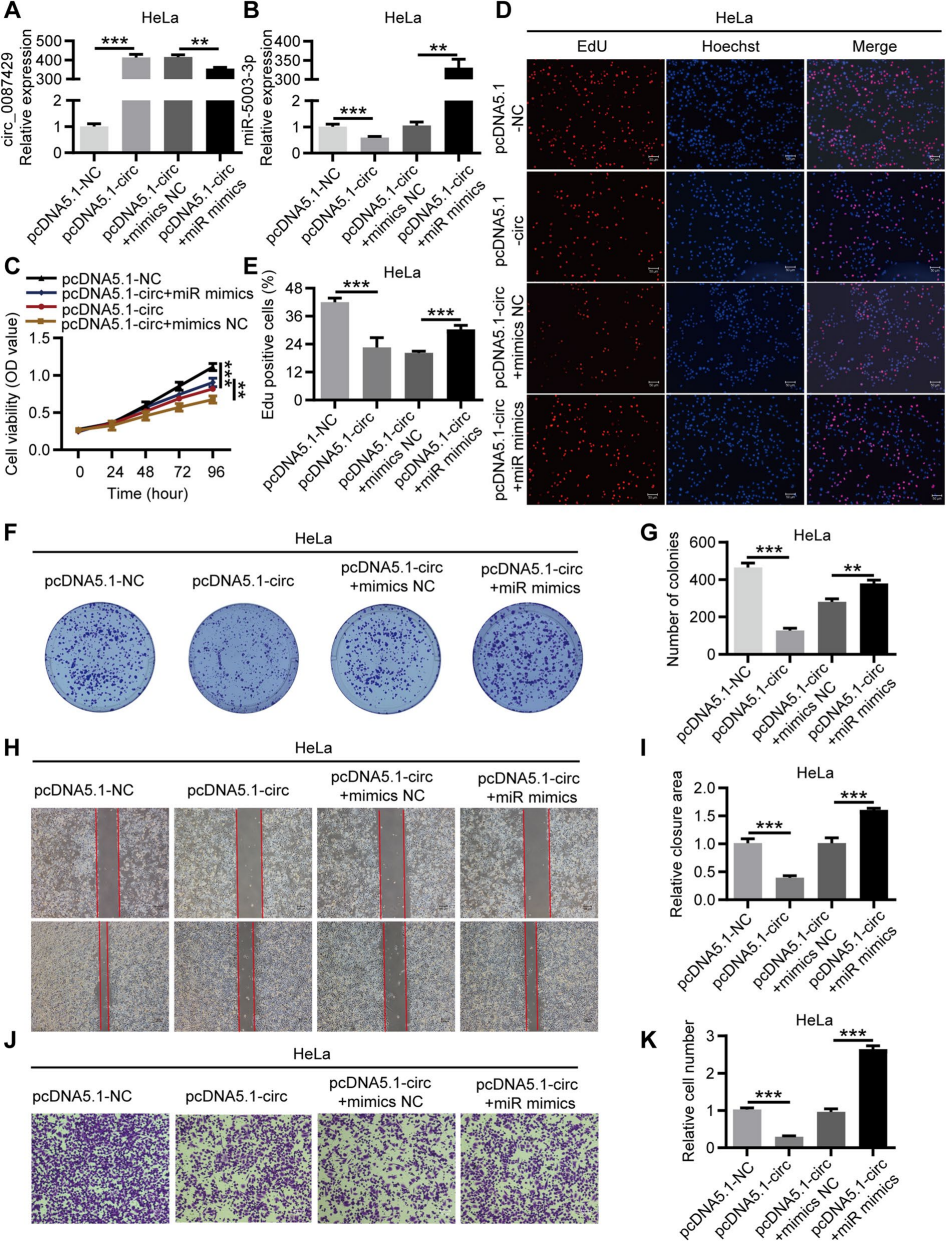
The tumour suppressor effect of circ_0087429 in cervical cancer can be reversed by miR-5003-3p. HeLa cells were transfected with pcDNA5.1-NC, pcDNA5.1-circ, pcDNA5.1-circ + mimics NC or pcDNA5.1-circ + miR mimics for subsequent detection. **a-b.** The expression of circ_0087429 and miR-5003-3p in each group was tested by qRT-PCR. **c-g.** CCK-8, EdU and colony formation assays were used to detect the proliferation ability of each group of cells. Scale bar, 50 μm. **h-i.** Wound healing assays determined the migration ability of each group of cells. Scale bar, 100 μm. **j-k.** Transwell invasion assays detected the invasion ability of each group of cells. Scale bar, 50 μm. **p < 0.01, ***p < 0.001

To verify whether circ_0087429 regulates the progression of cervical cancer through miR-5003-3p, we designed rescue experiments. HeLa cells were cotransfected with pcDNA5.1-circ_0087429 and miR-5003-3p mimics, qRT-PCR was used to verify the expression of circ_0087429 and miR-5003-3p after transfection (Fig. 5a-b), and the biological function of each group of cells was evaluated (Fig. 5c-k). Similarly, SiHa cells were cotransfected with si-circ_0087429 and miR-5003-3p inhibitor, and the biological function of each group of cells was evaluated (Fig. S1). The results showed that miR-5003-3p could significantly reverse the inhibitory effect of circ_0087429 on the proliferation, migration and invasion of cervical cancer cells. In conclusion, circ_0087429 can inhibit tumour progression by acting as a molecular sponge of miR-5003-3p in cervical cancer.

### OGN is a direct target gene of miR-5003-3p and is downregulated in CC

**Fig. 6:**
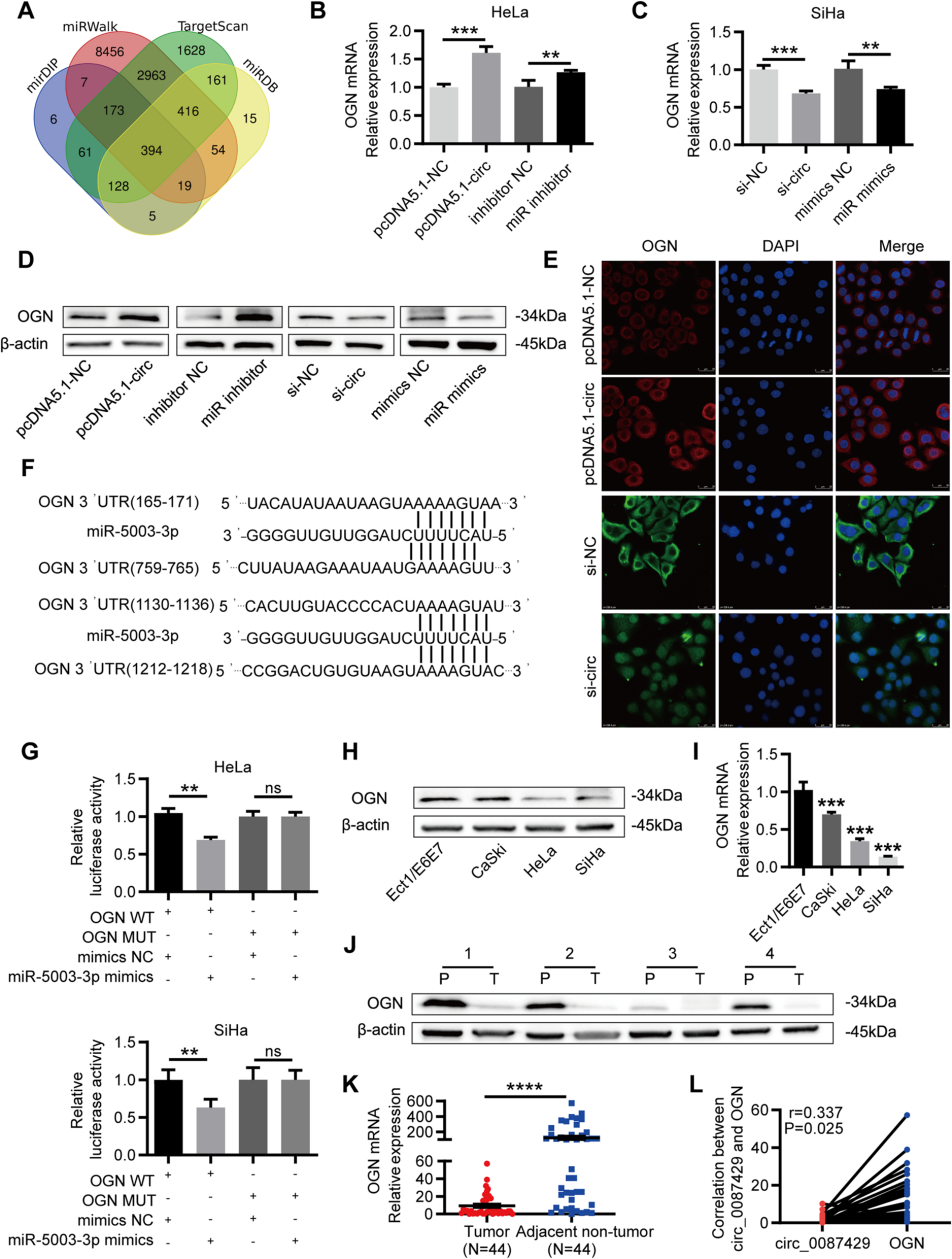
circ_0087429 downregulates the expression of OGN through competitive binding with miR-5003-3p in cervical cancer. **a.** The databases miRDB, miRWalk, mirDIP and TargetScan predicted the target genes of miR-5003-3p **b.** The expression changes in OGN after circ_0087429 overexpression and miR-5003-3p knockdown were determined by qRT-PCR. **c.** The expression changes in OGN after circ_0087429 knockdown and miR-5003-3p overexpression were determined by qRT-PCR. **d.** The expression of OGN was detected by western blotting after the expression of circ_0087429 and miR-5003-3p was changed. **e.** The fluorescence intensity of OGN after circ_0087429 overexpression or knockdown was confirmed by immunofluorescence staining. Scale bar, 25 μm. **f.** The binding sites of circ_0087429 and miR-5003-3p were predicted by miRWalk. **g.** A luciferase reporter assay was used to examine the relative luciferase activity of OGN-WT and OGN-MUT. **h-i.** The differential expression of OGN between cervical cancer and normal cervical epithelial cell lines was detected by western blotting and qRT-PCR. **j-k.** The differential expression of OGN between cervical cancer (T) and adjacent tissue (P) samples was detected by western blotting and qRT-PCR. **l.** Spearman’s correlation analysis confirmed the correlation between circ_0087429 and OGN expression in cervical cancer tissue samples. **p < 0.01, ***p < 0.001, NS means no statistical significance

We used the online prediction tools miRDB [24], miRWalk [25], mirDIP [26] and TargetScan [27] to predict the potential target genes of miR-5003-3p. As shown in the Venn diagram (Fig. 6a), the four databases predicted 394 common target genes. These target genes were crossed with mRNAs that are downregulated by more than fivefold in cervical cancer from The Cancer Genome Atlas (TCGA) database, and 8 target genes were finally obtained (OGN, SCN7A, PGR, PTGER3, FGF7, ADAMTS5, LMO3 and ANK2). Among them, the target gene OGN was the most significantly downregulated in cervical cancer (Fig. S2). Based on the above results, we used qRT-PCR and western blotting to detect the corresponding expression changes in OGN after circ_0087429 and miR-5003-3p expression was altered in cervical cancer cells. The results showed that OGN expression was significantly increased after circ_0087429 overexpression and miR-5003-3p knockdown, while OGN expression was significantly downregulated after circ_0087429 knockdown and miR-5003-3p overexpression (Fig. 6b-d). The results of immunofluorescence staining also showed that the overexpression of circ_0087429 upregulated the expression of OGN, while the expression of OGN decreased correspondingly after circ_0087429 knockdown (Fig. 6e). Then, based on the binding sites of miR-5003-3p and OGN, we constructed OGN-WT and OGN-MUT plasmids. A dual-luciferase reporter assay showed that overexpression of miR-5003-3p significantly reduced the luciferase activity of OGN-WT, while the luciferase activity of OGN-MUT was not affected (Fig. 6f-g). These results indicated that there was a direct interaction between miR-5003-3p and OGN.

It is reported that the expression of OGN is reduced in many different tumours. We assessed the expression of OGN in cervical cancer cells and tissues, and the results confirmed that OGN was significantly downregulated in cervical cancer (Fig. 6h-k). The analysis of the relationship between OGN and the clinicopathological parameters of cervical cancer patients showed that compared with that in patients with tumours less than 4 cm, the expression of OGN was significantly reduced in cervical cancer patients with larger tumours (Table 1). Correlation analysis showed that the expression levels of OGN and circ_0087429 in cervical cancer tissue were positively correlated (Fig. 6l).

**Fig. 7:**
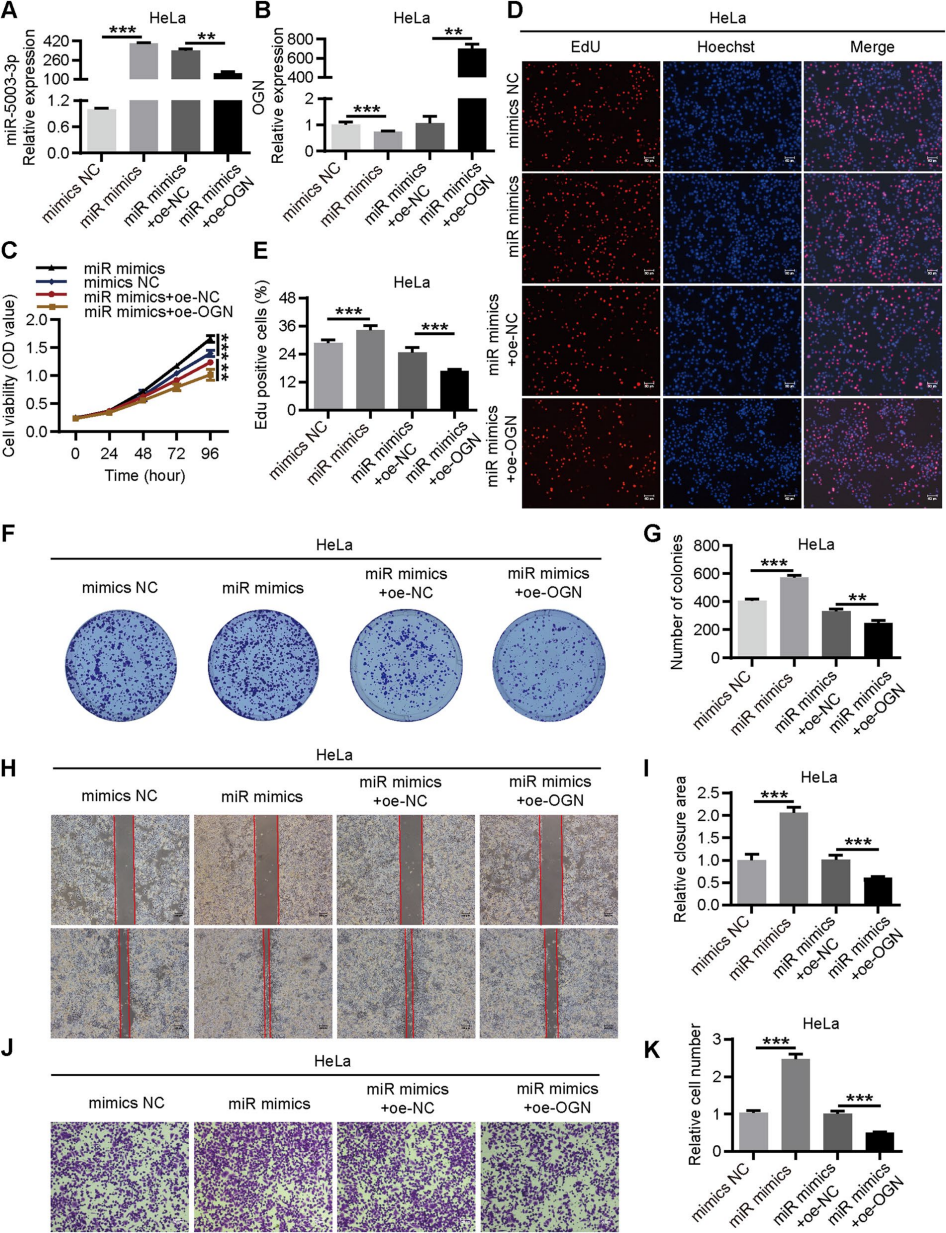
The cancer-promoting effect of miR-5003-3p in cervical cancer can be reversed by OGN. HeLa cells were transfected with mimics NC, miR mimics, miR mimics + oe-NC or miR mimics + oe-OGN for subsequent detection. **a-b.** The expression of miR-5003-3p and OGN in each group was tested by qRT-PCR. **c-g.** CCK-8, EdU and colony formation assays were used to detect the proliferation ability of each group of cells. Scale bar, 50 μm. **h-i.** Wound healing assays determined the migration ability of each group of cells. Scale bar, 100 μm. **j-k.** Transwell invasion assays detected the invasion ability of each group of cells. Scale bar, 50 μm. **p < 0.01, ***p < 0.001

After that, we constructed an overexpression vector of OGN and designed rescue experiments. HeLa cells were co-transfected with miR-5003-3p mimics and OGN overexpression plasmid (Fig. 7a-b), and the biological function of cells in each group was evaluated. The results showed that the promotion on the proliferation, migration and invasion of cervical cancer cells caused by miR-5003-3p mimics could be reversed by OGN overexpression (Fig. 7c-k). Similarly, HeLa cells were cotransfected with pcDNA5.1-circ_0087429 and si-OGN (Fig. S3a-b). The results showed that si-OGN could significantly reverse the inhibitory effect of circ_0087429 on the proliferation, migration and invasion of cervical cancer cells (Fig. S3c-k), indicating that the tumour suppressor effect of circ_0087429 was achieved through the miR-5003-3p/OGN axis.

### Circ_0087429 can inhibit EMT in cervical cancer cells through the circ_0087429/miR-5003-3p/OGN axis

**Fig. 8:**
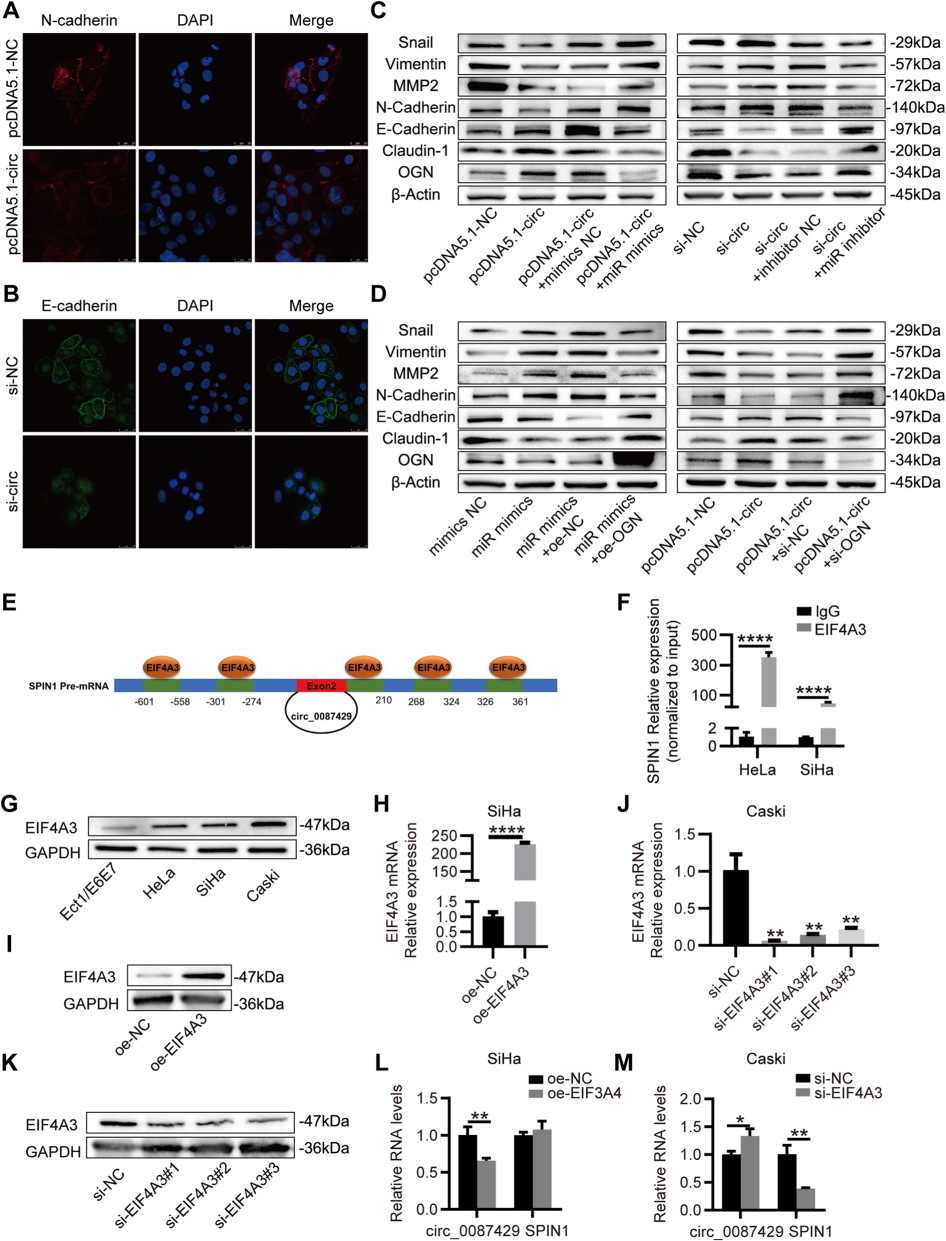
EIF4A3-regulated circ_0087429 can inhibit EMT in cervical cancer cells through the circ_0087429/miR-5003-3p/OGN axis**. a-b.** Immunofluorescence staining detected the fluorescence intensities of N-cadherin or E-cadherin upon circ_0087429 overexpression or knockdown, respectively. Scale bar, 25 μm. **c-d.** EMT related proteins in each group of cells were detected by western blotting. **e.** The binding sites for EIF4A3 in the flanking sequences of the SPIN1 mRNA transcript were predicted using CircInteractome. **f.** RIP assay demonstrated the binding between SPIN1 and EIF4A3. **g.** The differential expression of EIF4A3 between cervical cancer and normal cervical epithelial cell lines was detected by western blotting. **h-i.** The expression of EIF4A3 in the two groups of SiHa cells of oe-NC and oe-EIF4A3 was detected by qRT-PCR and western blotting. **j-k.** The expression of EIF4A3 in Caski cells treated with siRNA was detected by qRT-PCR and western blotting. **l.** The expression of circ_0087429 and EIF4A3 in SiHa cells stably overexpressing EIF4A3 were detected by qRT-PCR. **m.** The expression of circ_0087429 and EIF4A3 in Caski cells treated with si-EIF4A3 were detected by qRT-PCR. *p < 0.05, **p < 0.01, ****p < 0.0001

It is reported that miR-5003-3p and OGN may participate in the EMT of tumours. To this end, we assessed the effect of circ_0087429 on EMT related proteins. Immunofluorescence staining showed that after circ_0087429 overexpression, the expression of N-cadherin was downregulated, while knockdown of circ_0087429 decreased the expression of E-cadherin (Fig. 8a-b). The western blotting results showed that the overexpression of circ_0087429 increased the expression of E-cadherin, Claudin-1 and decreased the expression of N-cadherin, Vimentin, MMP2 and Snail, while overexpression of miR-5003-3p or knockdown of OGN both reversed the inhibitory effect of circ_0087429 on EMT. In addition, the promotion of EMT after circ_0087429 knockdown could also be reversed by the miR-5003-3p inhibitor, and overexpression of OGN also inhibited the promotion of EMT after miR-5003-3p overexpression (Fig. 8c–d). Therefore, the inhibitory effect of circ_0087429 on EMT is achieved through the circ_0087429/miR-5003-3p/OGN axis.

### EIF4A3 regulates the expression of circ_0087429

To explore how circ_0087429 is differentially expressed in cervical cancer, we used CircInteractome (https://circinteractome.nia.nih.gov/) to predict the RNA binding proteins that match the flanking regions of the circ_0087429, of which EIF4A3 has the most binding sites (Fig. 8e). The results of the RIP assay showed that the parental gene SPIN1 was significantly enriched in the anti-EIF4A3 group compared with the anti-IgG group (Fig. 8f). EIF4A3 was significantly upregulated in cervical cancer (Fig. 8g). After the overexpression of EIF4A3, the expression of circ_0087429 decreased significantly (Fig. 8h-i and 8l). Knockdown of EIF4A3 inhibited the expression of parental gene SPIN1, but significantly promoted the expression of circ_0087429 (Fig. 8j-k and 8m). In conclusion, EIF4A3 can inhibit circ_0087429 expression by binding to its flanking regions.

### Overexpression of circ_0087429 in vivo can inhibit the progression of cervical cancer

**Fig. 9:**
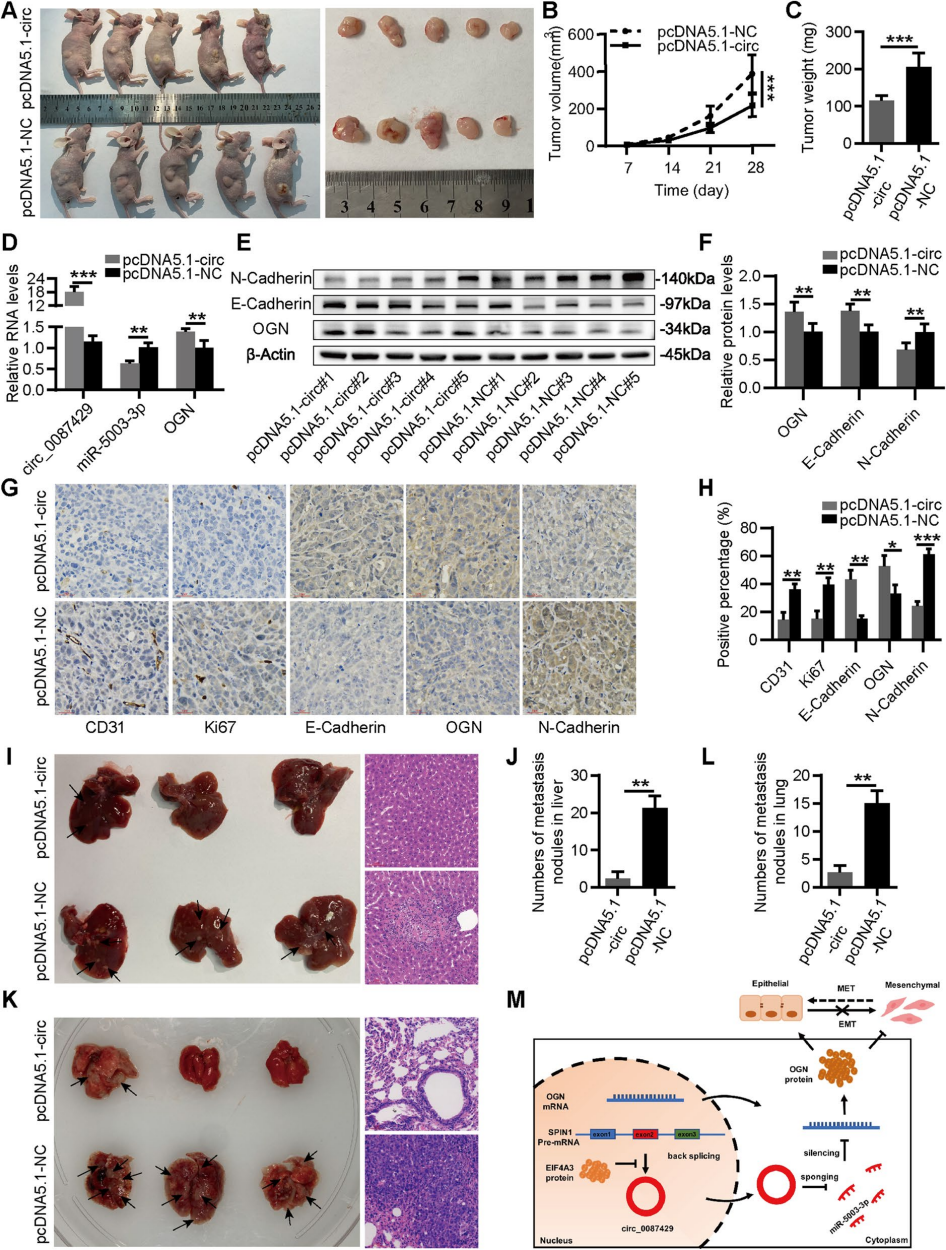
Overexpression of circ_0087429 in vivo can inhibit the progression of cervical cancer **a.** Image of subcutaneous tumour tissues in the circ_0087429-overexpressing group and control group. **b-c.** Volume and weight of subcutaneous tumour tissues in each group. **d.** The expression levels of circ_0087429, miR-5003-3p and OGN in subcutaneous tumour tissues of each group were detected by qRT-PCR. **e–f.** The expression levels of OGN, N-cadherin and E-cadherin in subcutaneous tumour tissues of each group were detected by western blotting. **g-h.** The expression levels of OGN, N-cadherin, E-cadherin, Ki67 and CD31 in subcutaneous tumour tissues of each group were detected by immunohistochemical staining. Scale bar, 30 μm. **i-l.** Representative images and bar graphs of liver (i-j) and lung (k-l) metastases with circ_0087429-overexpressing group and control group in a nude mouse metastatic tumour model. Metastatic nodules are indicated by arrows. Scale bar, 60 μm. **m.** A schematic diagram showing how circ_0087429 regulates the expression of OGN by sponging miR-5003-3p and thereby reverses EMT. MET: mesenchymal to epithelial transition. *p < 0.05, **p < 0.01, ***p < 0.001

To further examine the effect of circ_0087429 on tumour growth in animal models, we subcutaneously injected HeLa cells stably transfected with pcDNA5.1-NC or pcDNA5.1-circ_0087429 into BALB/c nude mice. Tumour size was measured every 7 days, and the mice were sacrificed after four weeks. The results showed that the tumour volume and weight were significantly reduced after circ_0087429 overexpression (Fig. 9a-c). The qRT-PCR results of subcutaneous tumour tissues showed that the expression of miR-5003-3p was downregulated after the overexpression of circ_0087429, while the expression of OGN was upregulated (Fig. 9d). The western blotting and immunohistochemical staining results showed that the expression of OGN and E-cadherin was increased, and the expression of N-cadherin was decreased in tumour tissues overexpressing circ_0087429. In addition, the expression of CD31 and Ki67 decreased in tumour tissues overexpressing circ_0087429 (Fig. 9e-h). In order to investigate the effect of circ_0087429 on tumour metastasis, we injected HeLa cells stably transfected with pcDNA5.1-NC or pcDNA5.1-circ_0087429 into nude mice through the tail vein. The results showed that the circ_0087429 overexpression group had fewer lung and liver metastatic nodules (Fig. 9i-l).

In summary, EIF4A3-downregulated circ_0087429 is a tumour suppressor gene that can promote the expression of OGN and inhibit the EMT of cervical cancer cells by competitively binding miR-5003-3p (Fig. 9m).

## Discussion

As a noncoding RNA, circRNAs are highly expressed, stable in structure, and able to survive in a complex microenvironment. They are considered to be key regulators of the occurrence and development of cancer [28]. In cervical cancer, some circRNAs with cancer-promoting effects, such as circTPCN [13] and other circRNAs with anticancer effects, such as circTPCN, have been reported [29]. However, the role of most circRNAs in the development of cervical cancer remains unexplored, and these circRNAs may provide new targets for clarifying the pathogenesis and refining the clinical treatment of cervical cancer [30]. In this study, we found that an unknown circRNA, circ_0087429, was significantly downregulated in cervical cancer. And circ_0087429 was closely related to the International Federation of Gynecology and Obstetrics (FIGO) stage and lymphatic metastasis of cervical cancer patients.

Based on this phenomenon, we explored the possible role and mechanism of circ_0087429 in cervical cancer. The results of functional experiments showed that circ_0087429 can significantly inhibit the proliferation, migration, invasion and angiogenesis of cervical cancer. The ceRNA mechanism is an important mechanism by which circRNAs function. The circRNAs in the cytoplasm can bind to miRNAs and inhibit their functions, thereby releasing downstream target genes from miRNA-mediated inhibition, highlight their roles as miRNA sponges [31]. This study first verified the ring structure of circ_0087429 and clarified its cellular location, which indicated that circ_0087429 could be involved in posttranscriptional regulation. After that, we used bioinformatics methods to screen out miR-5003-3p, which has a complementary sequence to circ_0087429, and a target gene of miR-5003-3p (OGN). qRT-PCR verified the correlations between the three genes. A dual-luciferase reporter assay was applied to verify the interactions between circ_0087429 and miR-5003-3p and between miR-5003-3p and OGN. The role of miR-5003-3p and OGN in cervical cancer is still unknown. This study verified the tumour-promoting effect of miR-5003-3p in cervical cancer for the first time and confirmed that it can block the tumour-suppressive effect of circ_0087429. Later, it was verified that OGN expression is downregulated in cervical cancer and is closely related to tumour size in cervical cancer patients. Functional experiments showed that OGN has a tumour-suppressive effect, the tumour-promoting effect of miR-5003-3p in cervical cancer can be inhibited by the target gene OGN, and downregulation of OGN can also block the tumour-suppressive effect of circ_0087429. The above results suggest that the tumour-suppressive effect of circ_0087429 in cervical cancer is achieved through the circ_0087429/miR-5003-3p/OGN axis.

EMT is the process by which quiescent epithelial cells transform into mesenchymal-phenotype cells. Cancer cells have plasticity and can continuously adapt to the constantly changing tumour microenvironment, and this process is mediated by EMT [32]. In addition, EMT is involved in cancer cell stemness regulation, metabolic reprogramming, treatment resistance, immune escape and other biological processes [33–35]. Studies have shown that miR-5003-3p in breast cancer can promote the stability of snail, and then promote tumour metastasis through EMT [17]. OGN can reverse EMT through the PI3K/Akt/mTOR pathway in breast cancer [22], and reduce the expression of ZEB-1 through the EGFR/Akt signaling pathway in colon cancer to inhibit EMT [21]. Given that both miR-5003-3p and OGN participate in EMT of tumours, we explored the influence of circ_0087429 on the EMT in cervical cancer. The results showed that circ_0087429 can inhibit EMT, and the inhibitory effect can be achieved through the circ_0087429/miR-5003-3p/OGN axis.

The exon junction complex (EJC) is deposited by spliceosomes onto mRNAs at 20–24 nucleotides upstream of exon-exon junctions, where it serves as a molecular marker for the correct splicing of precursor mRNA [36]. EIF4A3 is the core component of EJC, which can be used as RNA binding protein to play an important role by participating in mRNA splicing and influencing its downstream events such as transport and translation [37]. circRNAs are produced by alternative splicing [38]. Studies have reported that EIF4A3 can affect the expression level of circRNA by participating in the backsplicing of circRNA [39]. For example, EIF4A3 can be combined with the flanking sequence of circMMP9 to promote the expression of circMMP9 [40]. EIF4A3 is upregulated in various malignant tumours such as pancreatic cancer, breast cancer and cervical cancer [41–43]. In this study, we confirmed the binding of EIF4A3 to SPIN1 by RIP assay, and verified the expression changes of circ_0087429 and its parent gene SPIN1 after overexpression and knockdown of EIF4A3. In conclusion, EIF4A3 can inhibit circ_0087429 expression by binding to SPIN1 and affecting its cyclization. EIF4A3 plays a significant role in the induction and maintenance of stress granules [44]. The formation of stress granules provides the basis for tumours to exhibit an aggressive phenotype and drug resistance [45]. Accumulation of stress granules can also induce metabolic changes in cancer cells and promote tumour growth [46, 47]. It has been reported that circRNAs can participate in the formation of stress granules [48], while circRNAs play important roles in cancer metastasis, drug resistance and regulation of cancer cell metabolism [6, 49]. Does circ_0087429 play a role in the formation of stress granules in cancer cells? Can it participate in the metastasis, metabolism and drug resistance of cancer cells by regulating the formation of stress granules? These questions are all worthy of further exploration.

## Conclusions

In summary, our research shows that there is a new tumour suppressor gene, circ_0087429, in cervical cancer; circ_0087429 can regulate the expression of its target gene OGN by competitively binding with miR-5003-3p, thereby inhibiting EMT in the occurrence and development of cancer. EIF4A3 can inhibit its expression by binding to the flanking regions of circ_0087429. Our findings provide a new potential target for the treatment of cervical cancer.

## Supplementary Information

**Additional file 1: Table S1**. Primers used in this study. **Table S2**. Oligonucleotides used in this study. **Additional file 2**: **Fig. S1** The cancer-promoting effect of si-circ_0087429 in cervical cancer can be reversed by the miR-5003-3p inhibitor. SiHa cells were transfected with si-NC, si-circ, si-circ + inhibitor NC or si-circ + miR inhibitor for subsequent detection. a-b. The expression of circ_0087429 and miR-5003-3p in each group was tested by qRT–PCR. c-g. CCK-8, EdU and colony formation assays were used to detect the proliferation ability of each group of cells. Scale bar, 50 μm. h-i. Wound healing assays were used to determine the migration ability of each group of cells. Scale bar, 100 μm. j-k. Transwell invasion assays were used to detect the invasion ability of each group of cells. Scale bar, 50 μm. **p < 0.01, ***p < 0.001 .**Additional file 3: Fig. S2** The expression of 8 target genes in cervical tissues based on the GEPIA database. a. OGN, b. SCN7A, c. PGR, d. PTGER3, e. FGF7, f. ADAMTS5, g. LMO3, h. ANK2. *p < 0.05 .**Additional file 4: Fig. S3** The tumour suppressor effect of circ_0087429 in cervical cancer can be reversed by si-OGN. HeLa cells were transfected with pcDNA5.1-NC, pcDNA5.1-circ, pcDNA5.1-circ + si-NC or pcDNA5.1-circ + si-OGN for subsequent detection. a-b. The expression of circ_0087429 and OGN in each group was tested by qRT–PCR. c-g. CCK-8, EdU and colony formation assays were used to detect the proliferation ability of each group of cells. Scale bar, 50 μm. h-i. Wound healing assays were used to determine the migration ability of each group of cells. Scale bar, 100 μm. j-k. Transwell invasion assays were used to detect the invasion ability of each group of cells. Scale bar, 50 μm. **p < 0.01, ***p < 0.001, ****p < 0.0001 

## Data Availability

The datasets used and/or analysed during the current study are available from the corresponding author on reasonable request.

## Abbreviations

circRNA: Circular RNA
GEO: Gene Expression Omnibus
ceRNA: Competitive endogenous RNA
miRNA: MicroRNA
EMT: Epithelial to mesenchymal transition
SLRPs: Small leucine-rich proteoglycans
OGN: Osteoglycin
EIF4A3: Eukaryotic initiation factor 4A-III
qRT-PCR: Quantitative real-time polymerase reaction
FISH: Fluorescence in situ hybridization
CCK-8: Cell Counting Kit-8
EdU: 5-Ethynyl-20-deoxyuridine
IF: Immunofluorescence
IHC: Immunohistochemistry
SD: Standard deviation
FIGO: Federation International of Gynecology and Obstetrics
HPV: Human papilloma virus
TCGA: The Cancer Genome Atlas
ATCC: American Type Culture Collection
H&E: Hematoxylin and eosin
GEPIA: Gene Expression Profiling Interactive Analysis
EJC: Exon junction complex
RIP: RNA immunoprecipitation

## Declarations

### Author details

^1^Department of Gynecology, Shanghai First Maternity and Infant Hospital, School of Medicine, Tongji University, Shanghai 200092, China. ^2^Department of Gynecology, Shanghai East Hospital, School of Medicine, Tongji University, Shanghai 200120, China. ^3^Department of Obstetrics and Gynecology, Shanghai General Hospital, Shanghai Jiao Tong University School of Medicine, Shanghai 201620, China. ^4^Department of Gynecology, the First Affiliated Hospital of Wenzhou Medical University, Wenzhou, Zhejiang 325027, China.
