# Supplementary material for: EIF4A3-regulated circ_0087429 can reverse EMT and inhibit the progression of cervical cancer via miR-5003-3p-dependent upregulation of OGN expression
Source: J Exp Clin Cancer Res. 2022 May 5;41:165. doi: 10.1186/s13046-022-02368-4 (PMC9069757; doi:10.1186/s13046-022-02368-4)
Supplement: Supplementary file 1 — Additional file 1: Table S1. Primers used in this study. Table S2. Oligonucleotides used in this study. Additional file 2: Fig. S1 The cancer-promoting effect of si-circ_0087429 in cervical cancer can be reversed by the miR-5003-3p inhibitor. SiHa cells were transfected with si-NC, si-circ, si-circ + inhibitor NC or si-circ + miR inhibitor for subsequent detection. a-b. The expression of circ_0087429 and miR-5003-3p in each group was tested by qRT–PCR. c-g. CCK-8, EdU and colony formation assays were used to detect the proliferation ability of each group of cells. Scale bar, 50 μm. h-i. Wound healing assays were used to determine the migration ability of each group of cells. Scale bar, 100 μm. j-k. Transwell invasion assays were used to detect the invasion ability of each group of cells. Scale bar, 50 μm. **p < 0.01, ***p < 0.001 .Additional file 3: Fig. S2 The expression of 8 target genes in cervical tissues based on the GEPIA database. a. OGN, b. SCN7A, c. PGR, d. PTGER3, e. FGF7, f. ADAMTS5, g. LMO3, h. ANK2. *p < 0.05 .Additional file 4: Fig. S3 The tumour suppressor effect of circ_0087429 in cervical cancer can be reversed by si-OGN. HeLa cells were transfected with pcDNA5.1-NC, pcDNA5.1-circ, pcDNA5.1-circ + si-NC or pcDNA5.1-circ + si-OGN for subsequent detection. a-b. The expression of circ_0087429 and OGN in each group was tested by qRT–PCR. c-g. CCK-8, EdU and colony formation assays were used to detect the proliferation ability of each group of cells. Scale bar, 50 μm. h-i. Wound healing assays were used to determine the migration ability of each group of cells. Scale bar, 100 μm. j-k. Transwell invasion assays were used to detect the invasion ability of each group of cells. Scale bar, 50 μm. **p < 0.01, ***p < 0.001, ****p < 0.0001 [file 13046_2022_2368_MOESM1_ESM.pdf]

Table S1 Primer used in this study

Name	Primer sequences (5'-3')
circ_0087429	F: GGTCCAGAGCTGATGCAGAGTG
	R: GGTTAATCCACCATCCAAGTGTC
OGN	F: TCTACACTTCTCCTGTTACTGCT
	R: GAGGTAATGGTGTTATTGCCTCA
SPIN1	F: TGCTGAACTCGTAAAAGAGACAC
	R: GGTGTCTTTCCGAATGGGGT
EIF4A3	F: TGGCTCCCACAAGAGAGTTG
	R: GCACTGGACATTCATGTAGTCA
β-actin	F: CCTGTACGCCAACACAGTGC
	R: ATACTCCTGCTTGCTGATCC
GAPDH	F: CTGACTTCAACAGCGACACC
	R: TGCTGTAGCCAAATTCGTTGT
U6	F: CTCGCTTCGGCAGCACA
	R: AACGCTTCACGAATTTGCGT
miR-5003-3p	CGCGGCTACTTTTCTAGGTTG
miR-378i	AGGCGGACTGGACTAGGAGTC
miR-378a-3p	ACGCACTGGACTTGGAGTCA
miR-452-3p	CGGGCTCATCTGCAAAGAAG
miR-7846-3p	ATGCAGCGGAGCCTGGAG

Table S2 Oligonucleotides used in this study

Definition	sequences (5'-3')
si-circ_0087429-1	Sense: GAGCUGAUGCAGAGUGCUUGUTT<br>Antisense: ACAAGCACUCUGCAUCAGCUCTT
si-circ_0087429-2	Sense: GAUGCAGAGUGCUUGUGAUUUTT<br>Antisense: AAAUCACAAGCACUCUGCAUCTT
si-circ_0087429-3	Sense: UGCAGAGUGCUUGUGAUUUCATT<br>Antisense: UGAAAUCACAAGCACUCUGCATT
si-OGN-1	Sense: GACUCGAUUUUACAGGAAATT<br>Antisense: UUUCCUGUAAAAUCGAGUCTT
si-OGN-2	Sense: CAUUAUCUAUGAUUAUGGATT<br>Antisense: UCCAUAAUCAUAGAUAAUGTT
si-OGN-3	Sense: GGAAUCCGUGCCUCUUAAUUUTT<br>Antisense: AAAUUAAGAGGCACGGAUUCCTT
si-EIF4A3-1	Sense: GAGCAGAUUUACGAUGUAUTT<br>Antisense: AUACAUCGUAAAUCUGCUCTT
si-EIF4A3-2	Sense: CUCUCGGUGACUACAUGAATT<br>Antisense: UUCAUGUAGUCACCGAGAGTT
si-EIF4A3-3	Sense: CGAGCAAUCAAGCAGAUCATT<br>Antisense: UGAUCUGCUUGAUUGCUCGTT
si-NC	Sense: UUCUCCGAACGUGUCACGUTT<br>Antisense: ACGUGACACGUUCGGAGAATT

miR-5003-5p mimics	Sense: UACUUUUCUAGGUUGUUGGGG<br>Antisense: CCCCAACAACCUAGAAAAGUA
miR-5003-5p mimics NC	Sense: UCACAACCUCCUAGAAAGAGUAGA<br>Antisense: UCUACUCUUUCUAGGAGGUUGUGA
miR-5003-5p inhibitor	CCCCAACAACCUAGAAAAGUA
miR-5003-5p inhibitor NC	UCUACUCUUUCUAGGAGGUUGUGA
